# The spatial epidemiology of tuberculosis in Linyi City, China, 2005–2010

**DOI:** 10.1186/1471-2458-12-885

**Published:** 2012-10-19

**Authors:** Tao Wang, Fuzhong Xue, Yongjin Chen, Yunbo Ma, Yanxun Liu

**Affiliations:** 1 Department of Epidemiology and Health Statistics, Shandong University, Jinan, 250012, People’s Republic of China; 2 Zibo Municipal Center for Disease Control and Prevention, Zibo, 255026, People’s Republic of China; 3 Linyi People’s Hospital, Linyi, 276000, People’s Republic of China

## Abstract

**Background:**

Tuberculosis (TB) remains a major public health burden in many developing countries. China alone accounted for an estimated 12% of all incident TB cases worldwide in 2010. Several studies showed that the spatial distribution of TB was nonrandom and clustered. Thus, a spatial analysis was conducted with the aim to explore the spatial epidemiology of TB in Linyi City, which can provide guidance for formulating regional prevention and control strategies.

**Methods:**

The study was based on the reported cases of TB, between 2005 and 2010. 35,308 TB cases were geo-coded at the town level (n = 180). The spatial empirical Bayes smoothing, spatial autocorrelation and space-time scan statistic were used in this analysis.

**Results:**

Spatial distribution of TB in Linyi City from 2005 to 2010 was mapped at town level in the aspects of crude incidence, excess hazard and spatial smoothed incidence. The spatial distribution of TB was nonrandom and clustered with the significant Moran’s *I* for each year. Local *G*_i_^*^ detected five significant spatial clusters for high incidence of TB. The space-time analysis identified one most likely cluster and nine secondary clusters for high incidence of TB.

**Conclusions:**

There is evidence for the existence of statistically significant TB clusters in Linyi City, China. The result of this study may assist health departments to develop a better preventive strategy and increase the public health intervention’s effectiveness.

## Background

Tuberculosis (TB) is an infectious disease caused by the bacillus Mycobacterium tuberculosis, which is still a leading cause of death in low-income and middle-income countries, and remains a major public health burden in many developing countries [1]. China (0.9 million-1.2 million) alone accounted for an estimated 12% of all incident TB cases worldwide in 2010 [2]. Linyi City is one of the high-incidence areas in China, and there is an estimated 6,000 reported cases every year from 2005–2010. Thus, preventive strategies are urgently needed in the development of TB.

In recent years, geographical information systems (GIS) and spatial analysis were frequently used to describe the pattern of TB. In India, purely spatial and retrospective space-time analysis were used to find significant hotspots of TB in three areas of the Almora district [3]. In Japan, space-time scan statistics identified TB clusters in Fukuoka [4]. In Madagascar, spatial clustering of TB was associated with socio-economic and patient care factors in Antananarivo City [5]. With respect to spatiotemporal clustering, three high incidence space-time clusters were identified in Portugal between 2000 and 2004 [6]. In South African, GIS and spatial analysis were used to study TB transmission patterns in a high-incidence area [7]. In an Urban West African, spatial scan statistic was used to assess purely spatial and space-time clusters of TB in Greater Banjul [8]. Another study in Brazil investigated spatial patterns of the incidence of TB and its relationship with socio-economic status [9]. In Beijing, GIS and spatial analysis were used to determine the role of migration in the transmission of TB [10]. To our best knowledge, there were few studies to explore the spatial epidemiology of TB in Linyi City, China. A better understanding of the spatial epidemiology of TB may help health departments to provide guidance for formulating regional prevention and control strategies [9,10].

The spatial analyses, such as spatial smoothing, spatial autocorrelation and cluster analysis are commonly used to characterize spatial epidemiology of diseases. Spatial smoothing was used to reduce random variation associated with small populations [9,11-14]. Spatial autocorrelation analysis was performed to detect significantly difference from a random spatial distribution [10,15-17]. Spatial cluster analysis was conducted to identify whether cases of disease were geographically clustered [4,8,14,18-25].

Therefore, we conducted GIS-based spatial analyses involving spatial smoothing, spatial autocorrelation analysis and space-time scan statistic to characterize geographic distribution pattern of TB in Linyi City of China during 2005–2010.

## Methods

### Data collection and management

**Figure 1:**
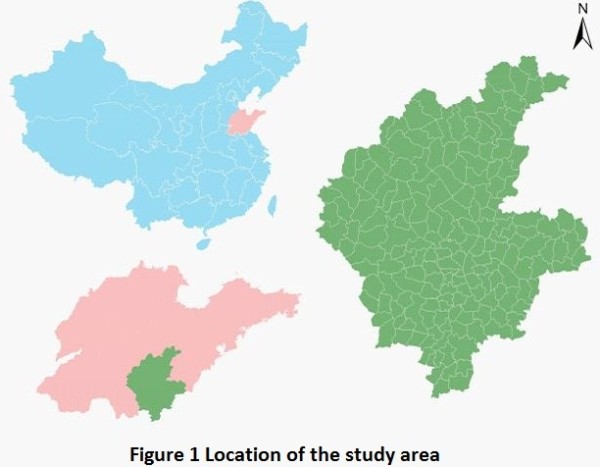
Location of the study area.

Relevant data for Linyi City (Figure 1), which is in the south of Shandong Province, was used in the study. Records on TB cases between 2005 and 2010 were obtained from the Linyi Institute for Tuberculosis Control, and we were permitted to use the data. To conduct a GIS-based analysis on the spatial distribution of TB, the town-level polygon map at 1:100,000 scale was obtained, on which the town-level point layer containing information regarding latitudes and longitudes of central points of each county was created. Demographic information based on Linyi Statistical Yearbook was integrated in terms of the administrative code. All TB cases were geo-coded and matched to the town-level layers of polygon and point by administrative code using the software ArcGIS9.3 (ESRI Inc., Redlands, CA, USA).

### GIS mapping and smoothing

To alleviate variations of incidence in small populations and areas, annualized average incidences of TB per 100,000 at each town over the 6 year-period were calculated, and spatial empirical Bayes smoothing was implemented in SpaceStat software [26].

To assess the risk of TB in each town, an excess hazard map was produced. The excess hazard represents the ratio of the observed incidence at each town over the average incidence of all areas, the later was calculated by the number of cases over the total number of people at risk instead of the annualized incidence of a town [27].

### Spatial autocorrelation analysis

Spatial autocorrelation analyses were performed in SpaceStat. Global Moran’s *I* statistics was used to discern spatial autocorrelation and detect the spatial distribution pattern of TB in Linyi City, China. Local *G*_i_^*^ was used to examine the local level of spatial autocorrelation and determine locations of clusters or hotspots. A calculated value of *G*_i_^*^ ≥1.96 indicates that the town and its neighboring towns have a TB incidence rate statistically significantly higher than other towns. The number of permutation test was set to 999 and the significance level was set as 0.05.

### Space-time scan statistic

The space-time scan statistic was performed using SaTScan^TM^ v9.1.1 software [28]. The method is defined by a cylindrical window with a circular geographic base and with height corresponding to time [29]. The null hypothesis assumed that the relative risk (RR) of TB was the same within the window compared to outside.

For this analysis, a Poisson based model was used, where the number of events in an area is Poisson distributed according to a known underlying population at risk [30]. The geographic size of the window was limited to half the expected number of cases and that the time size was limited to half the total time period [29]. The test of significance of the identified clusters was based on comparing the likelihood ratio test statistics against a null distribution obtained from Monte Carlo Simulation [19]. The number of permutation was set to 999 and the significance level was set as 0.05.

## Results

### Descriptive analysis of TB in Linyi City

**Figure 2:**
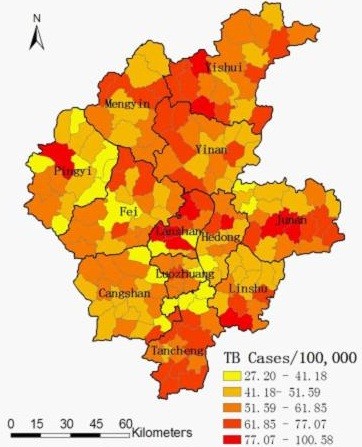
**Annualized average incidence of TB in Linyi City during 2005**–**2010.**

There were 35,329 TB cases reported in Linyi City, from 2005 to 2010. Of these, 35,308 (99.94%) had complete information including mapping of their place of residence. Annualized average incidence at the town-level ranged from 27.21 to 100.58 per 100,000 (Figure 2).

**Figure 3:**
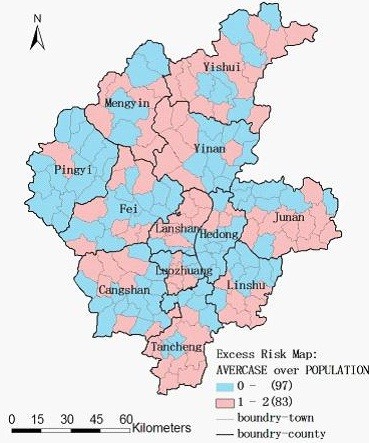
**Excess hazard map of TB in Linyi City**, **China from 2005 to 2010.**

The excess hazard map showed distribution of the excess risk, which was defined as a ratio of the observed number over the expected number of cases. Towns in blue color had lower incidences than expected, as indicated by excess risk values less than 1. In contrast, towns in red color had higher incidences than expected or excess risk values greater than 1 (Figure 3).

**Figure 4:**
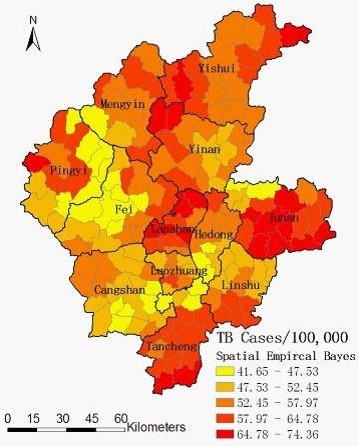
Spatially smoothed map of TB in Linyi City, China from 2005 to 2010.

Spatial empirical Bayes smoothed map for annualized average incidence was created by correcting the variance in the variability of incidence (Figure 4).

### Spatial autocorrelation analysis of TB in Linyi City

The global spatial autocorrelation analyses for annualized incidence of TB in Linyi City from 2005 to 2010 showed that the Moran’s *I* was significant for each year (Table 1), implying that distribution of TB was spatially autocorrelated in Linyi City, China.

**Table 1:** Global spatial autocorrelation analyses for annualized incidence of TB in Linyi City from 2005 to 2010

**Year**	**Moran’s*I***	***P*value**	**Pattern**
2005	0.694550	0.001	Clustered
2006	0.626996	0.001	Clustered
2007	0.573437	0.001	Clustered
2008	0.523944	0.001	Clustered
2009	0.449246	0.001	Clustered
2010	0.518774	0.001	Clustered

**Figure 5:**
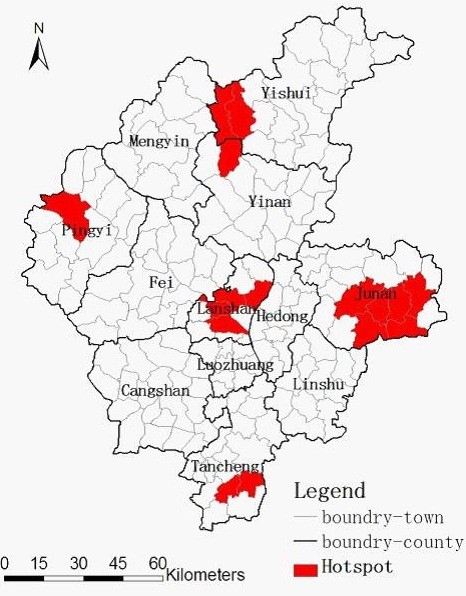
**Spatial clusters (hotspots) of the TB identified by local ***G*_I_^* ^**in Linyi City, China from 2005 to 2010.**

Five significant spatial clusters (hotspots) of TB were identified using the Local *G*_i_^*^ for spatial autocorrelation (Table 2 and Figure 5). The hotspots persisted in Pingyi Sub-district, Gaozhuang Town, Xiawei Town, Mamuchi Town, Pingshang Town, Zhuanggang Town, Fangqian Town, Xiangdi Town, Zhubian Town, Shizilu Sub-district, Gaofengtou Town, Guichang Town, Lanshan Sub-district, Baishabu Town, Yitang Town, and Zaogoutou Town from 2005 to 2010.

**Table 2:** **Spatial clusters (hotspots) of the TB identified by local ***G*_i_^* ^**in Linyi City, China from 2005 to 2010**

**Town**	***G***_i_^*^	***P*-value**	**Clusters**
Pingyi Sub-district	2.1975	0.028	Hotspot
Gaozhuang Town	2.9938	0.001	Hotspot
Xiawei Town	2.8946	0.048	Hotspot
Mamuchi Town	2.6845	0.027	Hotspot
Pingshang Town	2.8557	0.016	Hotspot
Zhuanggang Town	3.8660	0.005	Hotspot
Fangqian Town	3.3624	0.004	Hotspot
Xiangdi Town	4.5037	0.006	Hotspot
Zhubian Town	3.4762	0.005	Hotspot
Shizilu Sub-district	4.8718	0.004	Hotspot
Gaofengtou Town	2.7618	0.027	Hotspot
Guichang Town	3.0776	0.008	Hotspot
Lanshan Sub-district	2.7226	0.03	Hotspot
Baishabu Town	2.4016	0.033	Hotspot
Yitang Town	3.1462	0.024	Hotspot
Zaogoutou Town	2.9522	0.006	Hotspot

### Space-time analysis of TB in Linyi City

**Figure 6:**
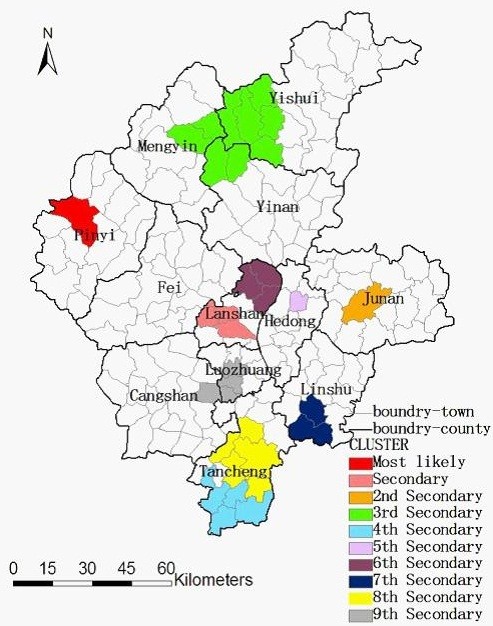
Space-time distribution of the detected clusters of TB cases with significant higher incidences in Linyi City, China from 2005 to 2010.

Space-time cluster analysis of TB in 2005–2010 in Linyi City showed that TB was not distributed randomly in space-time. The most likely statistically significant cluster for high incidence of TB was found to exist at Pingyi Sub-district, for the year 2008–2010 (RR = 2.22, p < 0.001), with 520 observed cases and 235.93 expected cases. Nine statistically significant secondary clusters were also detected for high incidence of TB. The results are listed in Table 3, and depicted on the map in Figure 6.

**Table 3:** SaTScan statistics for space-time clusters with significant higher incidence in Linyi City, China from 2005 to 2010

**Cluster type**	**Time frame**	**Cluster areas (n)**	**Observed cases**	**Expected cases**	**Relative risk**	***P*c p value**
Most likely	2008-2010	1	520	235.93	2.22	<0.001
Secondary	2007-2009	3	715	416.87	1.73	<0.001
2nd Secondary	2008-2010	1	358	185.34	1.94	<0.001
3rd Secondary	2008-2010	9	793	602.50	1.32	<0.001
4th Secondary	2006-2008	5	558	409.18	1.37	<0.001
5th Secondary	2005-2007	1	118	59.21	2.00	<0.001
6th Secondary	2007-2009	3	408	288.18	1.42	<0.001
7th Secondary	2007-2009	2	264	173.94	1.52	<0.001
8th Secondary	2008-2010	5	970	791.37	1.23	<0.001
9th Secondary	2008-2010	4	490	383.61	1.28	0.003

## Discussion

In our study, exploratory spatial data analysis and spatial cluster analysis of TB were conducted at town level in Linyi City, China. We mapped TB from different aspects such as crude incidence, excess risk, and spatial empirical Bayes smoothed incidence, investigated the spatial pattern and highlighted geographic areas with significant high incidence of TB in Linyi. The study showed that the spatial distribution of TB in Linyi City was nonrandom and clustered with the significant Moran’s *I* for each year. Local *G*_i_^*^ detected five significant spatial clusters for high incidence of TB when only space distribution was considered. However, one most likely cluster and nine secondary clusters for high incidence of TB were identified when both space and time were considered in the space-time analysis. When compared the clusters of the Local *G*_i_^*^ with those of the space-time scan statistic, both methods detected similar and significant high-risk clustering. Consistent results using these two methods, in addition to 6-years of TB case data, and a rate smoothing technique suggest that these results are robust.

The result of the present study provided useful information on the prevailing epidemiological situation of TB in Linyi City. The novel knowledge about the presence of clusters of TB in Linyi can help the Linyi Institute for Tuberculosis Control to intensify their remedial measures in the identified areas of high tuberculosis prevalence and chalk out future strategies for more effective TB control. Strategies may include compulsory BCG immunization of the children, educating the public about the dangers of TB as a re-emergent epidemic, and monitoring TB carefully.

While our study has demonstrated the usefulness of GIS and spatial analysis, it still has several limitations. First, our data relies on official surveillance and we cannot exclude the possibility that some towns may underreport the number of cases forvarious reasons. Cases might be missed by routine notification systems because people with TB do not seek care, seek care but remain undiagnosed, or are diagnosed by public and private providers that do not report cases to local or national authorities. Second, we analyzed a relatively short period of time (i.e. 6 years, from 2005 to 2010). Further studies are needed to evaluate the spatial and temporal changes in the pattern of TB using data from a longer study period. Third, we did not assess possible risk factors that could be associated with clustering. It is not a survey-based study but official surveillance, as socio-economic and environmental factors are not taken into account.

The present study only analyzed the statistically significant clusters of TB. Future researches are warranted to focus on the effect of various socio-economic and environmental factors on the high incidence of TB in the clustering areas. Disease prevalence is frequently associated with many aspects of socio-economic status, such as overcrowding [7], unemployment [7,31], low educational level [32,33], number of shebeens [7] and poor housing quality [34]. Moreover, spatial clustering of TB was also associated with the migrant population [10], patient care factors [5] and environmental factors [7]. After detecting the statistically significant clusters of TB in the region, a survey-based study is intended to identify the role of these factors in the spread of TB.

## Conclusion

This study showed the presence of clusters of TB in Linyi City, China and demonstrated that using the existing health data, the spatial statistic and GIS may provide public health officials with necessary feedback about the prevalence of statistically significant clusters of TB in the region, and thus enable them to carry out more effective strategies to control TB. Since the efficacy of TB control measures in specific areas could be assessed by a longitudinal change in TB prevalence, the space-time scan statistic also can contribute to health program evaluation. More detailed individual level investigations are needed in the identified clusters to evaluate the most important determinants of disease distribution.

## Competing interests

The authors declare that they have no competing interests.

## Authors’ contributions

TW extracted the data, conducted the statistical analysis and drafted the manuscript. FZX conceived of the project concept, helped to interpret the results and modify the manuscript. YJC helped to interpret the results. YBM extracted the data. YXL conceived of the project concept, assisted with the data interpretation, and helped write the manuscript. All of the authors have read and approved the final manuscript.

## Pre-publication history

The pre-publication history for this paper can be accessed here:

http://www.biomedcentral.com/1471-2458/12/885/prepub
